# MiR-204 down regulates SIRT1 and reverts SIRT1-induced epithelial-mesenchymal transition, anoikis resistance and invasion in gastric cancer cells

**DOI:** 10.1186/1471-2407-13-290

**Published:** 2013-06-14

**Authors:** Lihua Zhang, Xueqing Wang, Pingsheng Chen

**Affiliations:** 1 Department of Pathology, Southeast University, Zhongda Hospital, Nanjing 210009, P R China; 2 Department of Pathology, Southeast University, Nanjing 210009, P R China

## Abstract

**Background:**

Our previous studies showed that SIRT1 was over-expressed in gastric cancer specimens and related with lymph node metastasis. However, the mechanism of SIRT1 up-regulation and its association with metastasis in gastric cancer remain unclear. The present study was undertaken to understand the role of microRNA in regulation of SIRT1 in the progression of gastric cancer.

**Methods:**

Expression of miR-204 and SIRT1 was assessed in two gastric cancer cell lines and 24 matched cancer specimens. Luciferase reporter assay was carried to verify that miR-204 targeting SIRT1. Cell invasion ability of AGS and BGC was detected by transwell invasion assay. Annexin V/PI assay was used to investigate the cell sensitivity of anoikis. Western blot analysis to assess SIRT1, Vimentin, E-Cadherin, LKB1, and β-actin expression was performed in gastic cancer cell lines.

**Results:**

SIRT1 was defined as the target gene and elucidated the biological functions of miR-204 with a luciferase reporter assay and Western blot analysis. We verified that miR-204 levels were down-regulated and significantly associated with the up-regulation of SIRT1 mRNA levels in gastric cancer specimens. Over-expression of miR-204 reduced cell invasion and anoikis resistance in gastric cancer cells. Up-regulation of miR-204 influenced the levels of the epithelial mesenchymal transition (EMT)-associated genes, increasing E-cadherin levels and decreasing Vimentin levels. We demonstrated that the regulation of EMT by miR-204 involves cooperation with LKB1. Furthermore, silencing of SIRT1 phenocopied the effects of miR-204 in gastric cancer cells. These data demonstrate that miR-204 plays an important role in regulating metastasis of gastric cancer, which is involved in post-transcriptional repression of SIRT1.

**Conclusion:**

Our results suggest that down-regulation of miR-204 promotes gastric cancer cell invasion by activating the SIRT1-LKB1 pathway. These data demonstrate that miR-204 plays an important role in regulating metastasis of gastric cancer, which is involved in post-transcriptional repression of SIRT1.

## Background

Gastric cancer is among the most common malignancies in East Asian counties [1,2]. Recurrence and metastasis are the biggest obstacles for the treatment of gastric cancer [3]. Therefore, the search for new therapeutic targets to prevent the metastasis of gastric cancer is an urgent issue. However, the pathogenesis and mechanism underlying the metastasis process remain poorly understood. Epithelial-mesenchymal transition (EMT) is a key step toward cancer metastasis. Loss of E-cadherin expression is a hallmark of the EMT process and is likely required for enhanced tumor cell motility [4,5]. Epithelial cells lose epithelial characteristics and acquire mesenchymal characteristics by the down-regulation of E-cadherin [6].

Increasing evidence suggests that post-transcriptional regulation of gene expression, which is mediated by microRNAs (miRNAs), controls tumorigenesis and cancer metastasis [7-9]. Both the over-expression of oncogenic miRNAs and the decreased expression of tumor suppressor miRNAs play pivotal roles in cancer metastasis. Adam et al. demonstrated that miR-200 regulated EMT in bladder cancer cells and reversed resistance to epidermal growth factor receptor (EGFR) therapy [7]. This group also showed that the stable expression of miR-200 in mesenchymal UMUC3 cells increased E-cadherin levels; decreased protein expression of ZEB1, ZEB2, and ERRFI-1; decreased cell migration; and increased sensitivity to EGFR-blocking agents. Tie et al. described the regulation and function of miR-218 in gastric cancer metastasis. Decreased miR-218 levels eliminated Robo1 repression, which activated the Slit-Robo1 pathway through the interaction between Robo1 and Slit2 to trigger tumor metastasis [10].

In the current study, we investigated the role of miR-204 in gastric cancer metastasis. We demonstrated that the miR-204 expression was down-regulated in gastric cancer tissues and confirmed that the SIRT1 gene is the direct target of miR-204. Restoration of miR-204 or the knockdown of SIRT1 in metastatic gastric cancer cells induces a shift toward an epithelial morphology concomitant with increased expression of E-cadherin and decreased expression of Vimentin. Down-regulation of miR-204 inactivated LKB1 through SIRT1 to promote human gastric cancer cell invasion.

## Methods

### Cell lines and clinical samples

The AGS and BGC gastric cancer cell lines used in this study were cultured at 37°C in 5% CO_2_ and 95% air. All cells were grown in Dulbecco’s modified Eagle’s medium (Invitrogen, California, USA) supplemented with 1 mmol/L L-glutamine, 10% fetal bovine serum (Life Technologies, Inc., Burlington, Canada), penicillin G 100 U/mL, and streptomycin 100 mg/mL.

The Ethics Review Board of Zhongda Hospital, Southeast University Nanjing, China, approved this study. Informed consent was obtained from all patients. We studied gastric cancer specimens (cancer lesions and adjacent non-tumor mucosa) from 24 patients who had undergone resection at the Zhongda Hospital, Southeast University between 2005 and 2010. We gathered all samples in the same manner; they were snap-frozen immediately in liquid nitrogen and stored at −80°C until RNA extraction could be performed. All tissue specimens were evaluated pathologically. No patients had received irradiation or cancer chemotherapy prior to resection.

### RT-PCR and real-time RT-PCR

Total cellular RNA was extracted using Trizol (Invitrogen, California, USA). For mRNA detection, SIRT1, E-Cadherin, Vimentin and GAPDH mRNA expression were analyzed by the Sybr Green qRT-PCR according to the manufacturer’s instructions (Applied Biosystems).

For miRNA detection, polyA tail was added to RNase-free DNase digested total RNA using the E.coli polyA polymerase (NEB). Two micrograms of the tailed total RNA was reverse transcribed with ImProm-II (Promega). Conventional PCR or Sybr Green qRT-PCR was used to assay miRNA expression with the specific forward primers and the universal reverse primer complementary to the anchor primer. Anchor RT primer was used as the template for negative control and U6 as internal control.

The primers used are listed in Table 1.

**Table 1:** Sequence of RT-Primers

**Primers**	**Sequence(5’-3’)**
miR-138 F	agctggtgttgtgaatcaggccg
miR-155 F	ttaatgctaatcgtgataggggt
miR-181a F	aacattcaacgctgtcggtg
miR-181b F	aacattcattgctgtcggtgggt
miR-181c F	aacattcaacctgtcggtgagt
miR-181d F	aacattcattgttgtcggtgg
miR-30a F	gctgtaaacatcctcgactgga
miR-30b F	gccttgtaaacatcctacactcag
mIR-30c F	gtaaacatcctacactctcagc
miR-30d F	ctgtaaacatccccgactgg
miR-30e F	ccggtgtaaacatccttgactg
miR-204 F	ttccctttgtcatcctatgcct
miR-211 F	ttccctttgtcatccttcgcct
miR-9 F	tctttggttatctagctgtatga
miR-135a F	tatggctttttattcctatgtga
miR-135b F	tatggcttttcattcctatgtga
miR-133a F	tttggtccccttcaaccagctg
miR-133b F	tttggtccccttcaaccagcta
miR-22 F	cgtaagctgccagttgaagaa
miR-199a F	cccagtgttcagactacctgtt
miR-199b F	gtcccagtgtttagactatctgttc
miR-128 F	tcacagtgaaccggtctcttt
miR-217 F	tactgcatcaggaactgattgga
miR-200a F	ccctaacactgtctggtaacgat
miR-141 F	ggtaacactgtctggtaaagatgg
miR-34a F	tggcagtgtcttagctggttgt
Anchor RT primer	cgactcgatccagtctcagggtccgaggtattcgatcgagtcgcacttttttttttttv
Universal rev primer	ccagtctcagggtccgaggtattc
U6F	ctcgcttcggcagcaca
U6T	aacgcttcacgaatttgcgt
SIRT1 F	gccagagtccaagtttagaaga
SIRT1 T	ccatcagtcccaaatccag
E-Cadherin F	acagccccgccttatgatt
E-Cadherin T	tcggaaccgcttccttca
Vimentin F	tacaggaagctgctggaagg
Vimentin T	accagagggagtgaatccag
GAPDH F	gcaagttcaacggcacag
GAPDH T	cgccagtagactccacgac

### Luciferase reporter assay

Using Lipofectamine 2000 (Invitrogen), HEK293 cells (10^4^ cells/well) were plated in a 24-well plate. The cells were then co-transfected with 20 mM of either miR-204 or microRNA control, 40 ng of either pGL3-promoter-SIRT1-3’UTR-WT or pGL3-promoter-SIRT1-3’UTR-MUT, and 4 ng of pRL-TK (Promega, Madison, WI). HEK293 cells were collected 24 hours after transfection and analyzed using the Dual-Luciferase Reporter Assay System (Promega). The pRL-TK vector responsible for the constitutive expression of Renilla luciferase (Promega) was co-transfected as an internal control to correct for differences in both transfection and harvest efficiencies. Transfections were performed in triplicate and repeated at least three times in separate experiments.

### Western blot analysis and antibodies

Western blot analysis to assess SIRT1, Vimentin, E-Cadherin, LKB1, and β-actin expression was performed as previously described [11]. All of these primary antibodies were purchased from Santa Cruz Biotechnology (Santa Cruz, Daly City, CA).

### In vitro cell invasion assay

For transwell invasion assays, 1×10^5^ cells were placed on the non-coated membrane in the top chamber (CytoSelectTM 24 Well Cell Migration and Invasion Assay Combo Kit, 8-μm, CBA100-C, Cell Biolab, United States). Cells were plated in medium without serum. Medium supplemented with serum was used as a chemo-attractant in the lower chamber. The cells were incubated for 24 hours; cells that did not invade through the pores were removed using a cotton swab. Cells on the lower surface of the membrane were fixed with methanol and stained with crystal violet (Fisher Scientific Co., Fairlawn, NJ). The cell numbers were determined by counting the penetrating cells under a microscope at 200 magnification in random fields in each well. Each experiment was performed in triplicate.

### Anoikis assay

Poly-hydroxyethyl methacrylate (poly-HEMA, Sigma- Aldrich) was reconstituted in 95% ethanol to a concentration of 12 mg/mL. To prepare poly-HEMA coated plates, 0.5 mL of 12 mg/mL solution was added to each well of a 24-well plate and allowed to dry overnight in a laminar flow tissue culture hood. Cells were transfected as before. Twenty-four hours after transfection, 50,000 cells were plated in triplicate in poly-HEMA coated 24-well plates using regular culture medium. Following the addition to poly-HEMA coated plates, cells were collected at 2, 4, 8, 24 and 48 hrs post plating. Cell apoptosis was assayed by Annexin FITC/PI staining following manufacturer instructions (Invitrogen, California, USA). Briefly, cells were collected and washed in cold PBS. Cells were incubated for 15 min at room temperature in the presence of 1 μl Annexin V-FITC, 1 μl of propidium iodide and 98 μl of 1x binding buffer (all reagents provided by the manufacturer). After incubation, 400 μl of 1X binding buffer was added to each tube, and cells were analyzed by flow cytometry.

### Databases and statistics

We computationally screened target genes of miR-204 with the Target Scan program (http://www.targetscan.org/index.html), PicTar (http://pictar.bio.nyu.edu/), miRanda (http://www.microrna.org/microrna/home.do), miRBase (http://microrna.sanger.ac.uk) and microRNAMap (http://mirnamap.mbc.nctu.edu.tw).

We used the paired Wilcoxon nonparametric test to analyze pairs of non-tumor mucosa and cancer samples. The statistical significance of intergroup differences was determined using the χ^2^ test. All statistical analyses were performed using SPSS 16.0 software (SPSS, Chicago, IL). Differences were considered significant if P < 0.05. All experiments were performed in triplicate and repeated at least three times.

## Results

### Expression of miR-204 is significantly down-regulated in gastric cancer and associated with cancer metastasis

**Figure 1:**
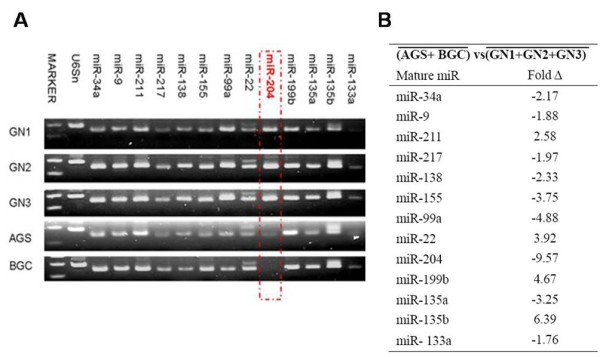
**Expression of miRNAs in gastric cancer cell lines (AGS and BCG) and 3 randomly selected normal gastric samples (GN1-GN3).** RT-PCR was carried out to determine the expression of microRNAs. Expression of miR-204 was the most significantly down regulated compared to the normal gastric samples (**A**). The average fold change of miRNAs calculated by qRT-PCR results are presented in the table (**B**).

**Figure 2:**
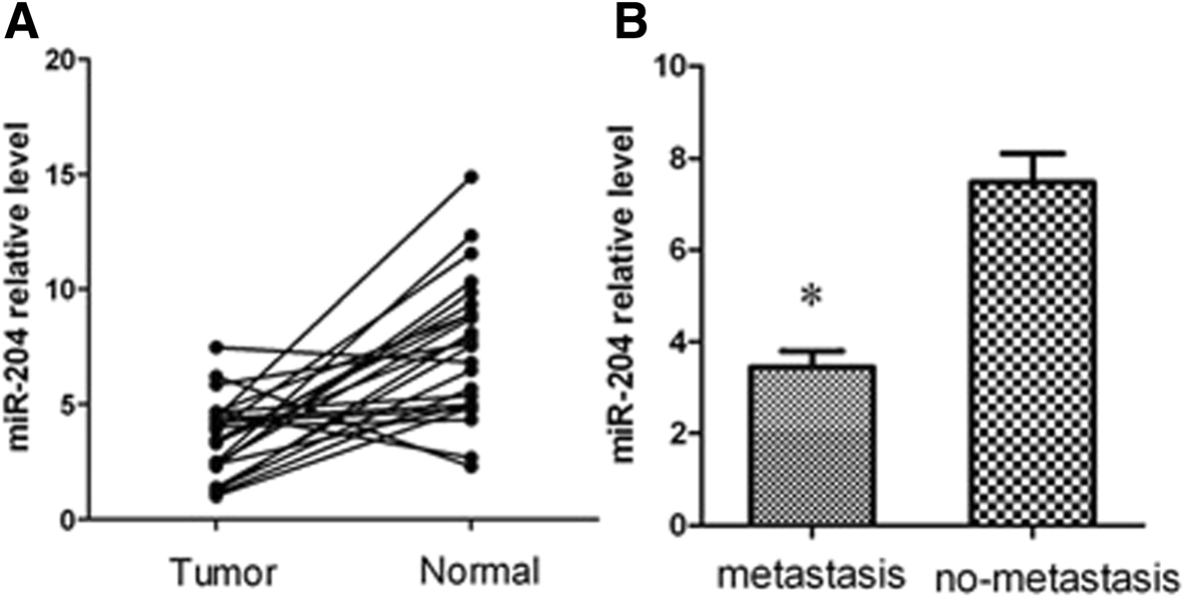
**MiR-204 is down-regulated in gastric tumor tissues.** (**A**) The analysis of the miR-204 expression level was performed in gastric tumor tissues (n = 24) and matched normal tissues. The miR-204 level was significantly down-regulated in gastric cancer tissues compared with that in matched normal tissues. (**B**) The gastric cancer samples were divided into two groups based on clinical progression. MiR-204 levels in the metastasis group (n = 14) were lower than those in the no-metastasis group. Total RNA was subjected to RT-PCR to analyze the expression level of miR-204 in each sample. U6 was used as a reference for miRNAs. Each sample was analyzed in triplicate. The standard curve method was used to quantify the relative gene expression levels. **P < 0.01. *P < 0.05.

The expression of all miRNAs conserved across various species and predicted to target SIRT1 through bioinformatics was evaluated. Evaluation in 3 normal gastric mucosa tissues, 2 gastric cancer cell lines was performed using Conventional RT-PCR (Figure 1A). QRT-PCR was also carried to investigate the differential expression profile of microRNAs in gastric cancer cell lines vs normal gastric mucosa tissues (Figure 1B). We confirmed that reduced expression of miR-204 in the 2 gastric cancer cell lines. The expression of miR-204 and SIRT1 mRNA in 24 gastric cancer tissues and the matched normal tissues were evaluated using qRT-PCR to assess the role of miR-204 and its association with SIRT1 expression in gastric cancer tissues. Figure 2A shows that the miR-204 levels were significantly down-regulated in gastric cancer tissues compared with their matched normal tissues (**P<0.01). The mean expression level of miR-204 was 1.4 and 8.5 in the gastric cancer and matched normal tissue, respectively. We found that miR-204 expression was remarkably down-regulated in 24 primary tumors stratified based on clinical progression that subsequently metastasized when compared to the primary tumors that did not recur (Figure 2B). These data indicate that down-regulation of miR-204 may be related to the onset of gastric cancer metastasis. Notably, we found that miR-204 down-regulation was significantly associated with up-regulation of SIRT1 mRNA levels in gastric cancer specimens (Table 2). N/T ratios were classified as high based on highest textiles. MiR-204 and SIRT1 expressions were then dichotomized as high and low. Therefore, SIRT1 may be the target of miR-204 in gastric cancer cells.

**Table 2:** Correlation between miR-204 expression and SIRT1 mRNA expression in 24 gastric carcinomas

		**miR-204 expression**		***P***
	**Case number**	**N/T>7.5**	**N/T≤7.5**	
Overall	24	17	7	
SIRT1 mRNA expression				0.038*
N/T≤2.5	18	15	3	
N/T>2.5	6	2	4	

*P<0.05.

### Interaction of miR-204 with the 3’UTR of SIRT1 mRNA

**Figure 3:**
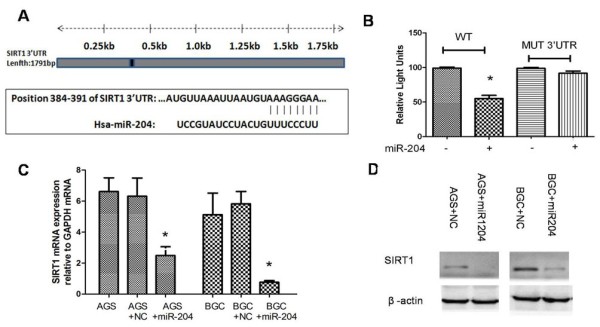
**SIRT1 is a new target for regulation by miR-204.** (**A**) illustration of SIRT1 3’UTR as well as the seed sequence of miR-204 showing the computationally predicted target region on the 3’UTR of SIRT1 mRNA. (**B**) miR-204 decreases SIRT1 3’UTR luciferase activity. HEK293T cells were transfected with 2μg of pGL3 luciferase vector containing either SIRT1 3’UTR or SIRT1 MUT 3’UTR (with an A-to-G mutation in miRNA-responsive element). Cells were co-transfected along with 50 nM miR-204 precursor for 24 h and then lysed and assessed for luciferase activity. Mean-S.E. (n =3). (**C**) SIRT1 mRNA levels in AGS and BGC transfected with 50 nM miR-204 precursor for 72 h. Mean-S.E. (n=3). (**D**) Western blotting of SIRT1 protein levels. SIRT1 levels in AGS and BGC transfected with 50 nM miR-204 precursor for 48 h (n=3). **P < 0.01. *P < 0.05.

The results presented so far demonstrate that the inactivation of miR-204 may be related to the up-regulation of SIRT1 in gastric cancer cells. Down-regulation of SIRT1 in gastric cancer cells may occur through the binding of miR-204 to the 3’UTR of SIRT1 mRNA. Target Scan (release 5.1) predicted a single miRNA-responsive element containing a conserved 8-mer exact seed match at positions 384–391 of SIRT1 3’-UTR as a miR-204 target (Figure 3A). To investigate this potential interaction, wild-type SIRT1 3’-UTR as well as mutSIRT1 3’-UTR with mutated target sites (A to G) were cloned into a pGL3 luciferase vector. To examine the impact of miR-204 on SIRT1 3’-UTR activity, HEK293T cells were co-transfected with miR-204 precursor (Ambion) that restored miR-204 expression. Figure 3B shows that miR-204 inhibits the luciferase activity of the wild-type SIRT1 3’-UTR, but mutation of the miR-204 miRNA-responsive element within the SIRT1 3’-UTR abolishes miR-204 action, suggesting that miR-204 targets one complementary sequence in the SIRT1 3’-UTR.

To determine whether miR-204 can affect endogenous SIRT1 expression, we examined the effect of miR-204 activation on SIRT1 in AGS and BCG cells. Real-time PCR and Western blots revealed significantly decreased expression of both SIRT1 mRNA and protein in GCCs transfected by miR-204 mimics (Figure 3C & D). These results indicate a negative role for miR-204 in the regulation of SIRT1 expression.

### Over-expression of miR-204 and down regulation of SIRT1 induces an mesenchymal -to- epithelial transition phenotype

**Figure 4:**
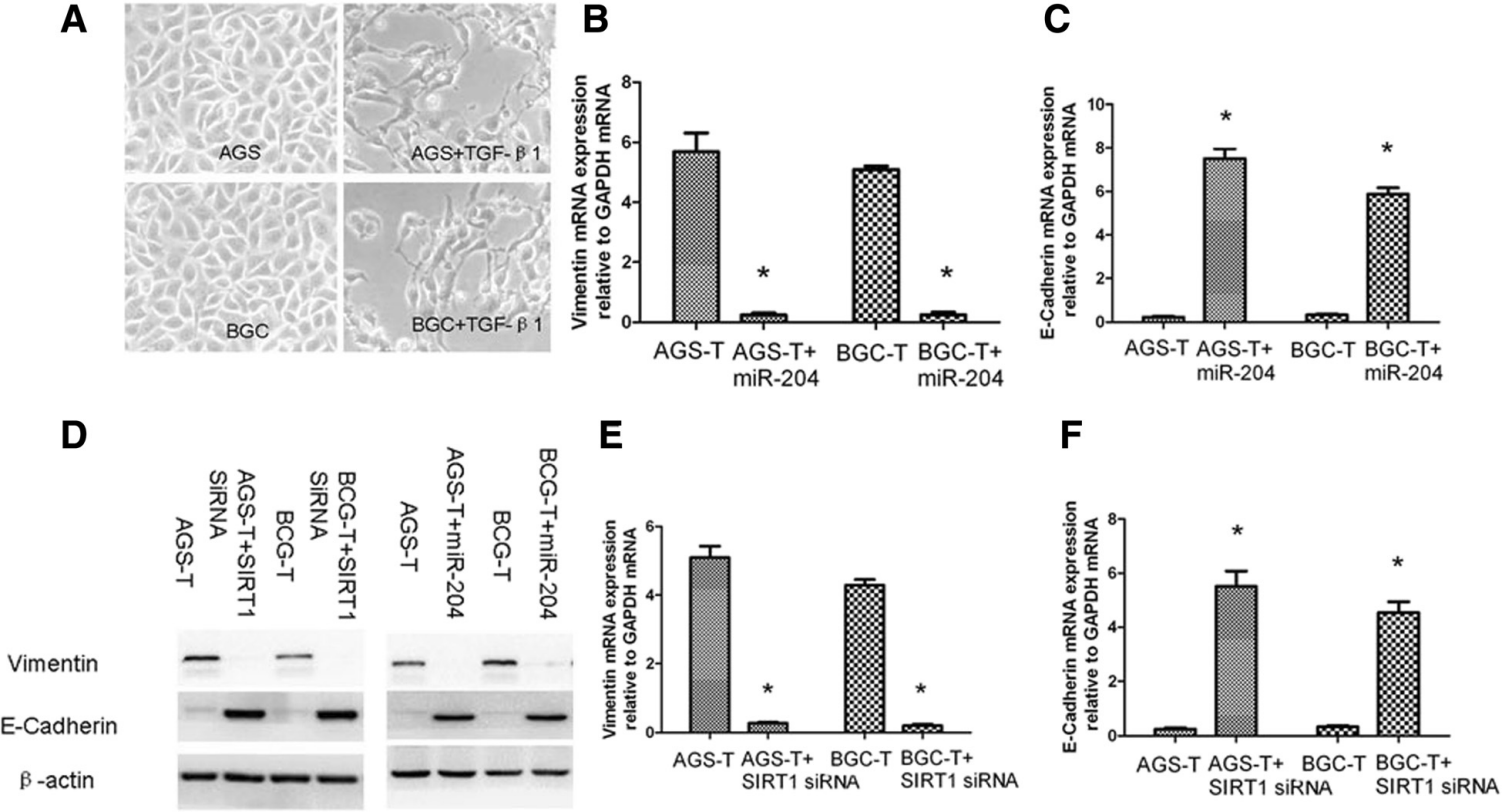
**Transformation of gastric cancer cells is associated with altered SIRT1 and miR-204 expression.** (**A**) An altered morphology is associated with transformation. Representative images display morphological changes from HGC to HGC-T (treated with 10 ng/ml TGF-β1for 21 days). Scale bar ,100μm. Vimentin (**B**) and E-cadherin (**C**) mRNA levels in transformed cells transfected with 50 nM miR-204 precusor for 72h. (**D**) Western bloting of E-cadherin and Vimentin protein levels. E-cadherin and Vimentin levels in HGC-T transfected with 50 nM miR-204 precusor or SIRT1 SiRNA for 48h. Vimentin (**E**) and E-cadherin (**F**) mRNA levels in transformed cells transfected with 50 nM SIRT1 SiRNA for 72h. Representative results from 3 independent experiments. Real-time RT-PCR results for mRNA levels were normalized to GAPDH mRNA. **P < 0.01. *P < 0.05.

We examined an in vitro model of EMT-like transformation and monitored alterations of SIRT1 and miR-204 expression. The immortalized cells were cultured in the presence of 10% FBS and treated with 10 ng/ml human TGF-β1 for 21 days. Treatment with TGF-β1 has been shown to induce EMT-like transformation of epithelial cells in many cell culture models (42). Figure 4A shows that treatment with TGF-β1 leads immortalized HGC cells (AGS and BCG cells) that undergo EMT-like transformation. This transformation is evidenced by loss of cell-cell adhesion and alterations of morphology from a round compact shape to a spindle shape. These transformed cells were defined as HGC-T. To define the role of miR-204 and SIRT1 in the progression of cell metastasis in gastric cancer cells, we treated the AGS-T and BGC-T cells with miR-204 mimics and SIRT1 SiRNA. Up-regulation of miR-204 levels and down regulation of SIRT1 was associated with the decrease of Vimentin mRNA transcripts (Figure 4B & E). The increase of E-cadherin mRNA transcripts was detected in AGS-T cells that overexpressed miR-204 or had SIRT1 knocked-down (Figure 4C & F). These differences were also true at the protein level, as shown for E-cadherin and Vimentin using Western blot analysis (Figure 4D).

### Over-expression of miR-204 and knock-down of SIRT1 inhibit gastric cancer cell metastasis

**Figure 5:**
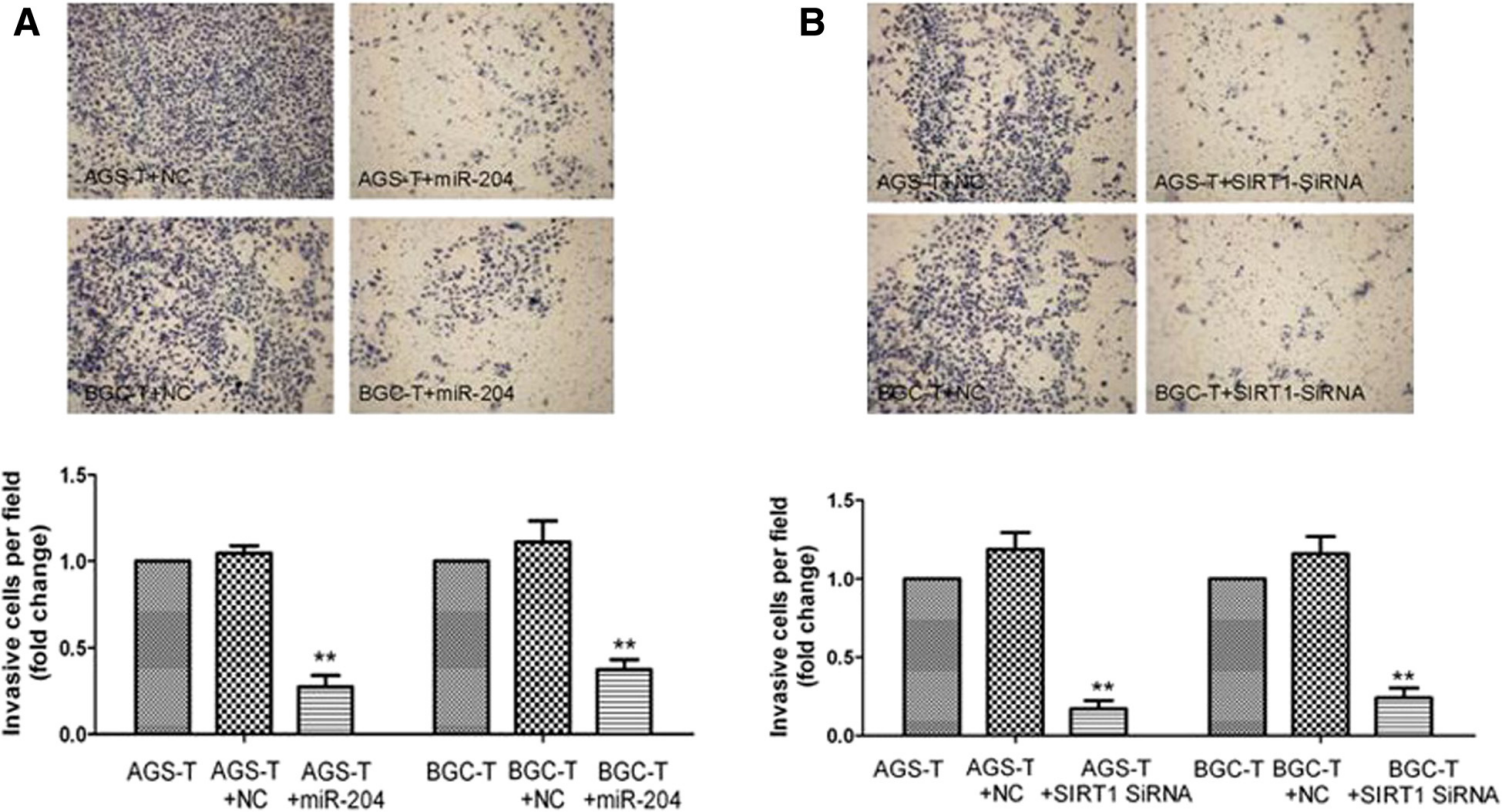
**MiR-204 promotes gastric cancer cell invasion.** (**A**) miR-204 was involved in AGS and BCG cell migration. The profiles and the images are representative of at least three independent experiments. (**B**) SIRT1 SiRNA inhibited AGS and BCG cell migration. The profiles and the images are representative of at least three independent experiments. N.C, negative control. *P < 0.05.

To study the physiological role of miR-204 and SIRT1 in metastasis, the gastric cancer cell lines treated with miR-204 mimics and SIRT1 SiRNA were analyzed. The ability of SIRT1 and miR-204 to regulate cell migration and cell invasion was investigated utilizing transwell invasion assays. HGC-T cells transfected with either SiRNA for SIRT1 (HGC-T/SiSIRT1) or miR-204 mimics (HGC-T /miR-204) showed dramatically decreased cell invasion (Figure 5). These findings further confirmed a functional role for SIRT1 in promoting EMT-like transformation of gastric cancer epithelial cells as well as establishing an inhibitory role for miR-204 in this process. These data suggest that miR-204 negatively regulates and SIRT1 positively regulates gastric cancer cell metastasis.

### Restoration of miR-204 and down-regulation of SIRT1 mediate suppression of anoikis resistance

**Figure 6:**
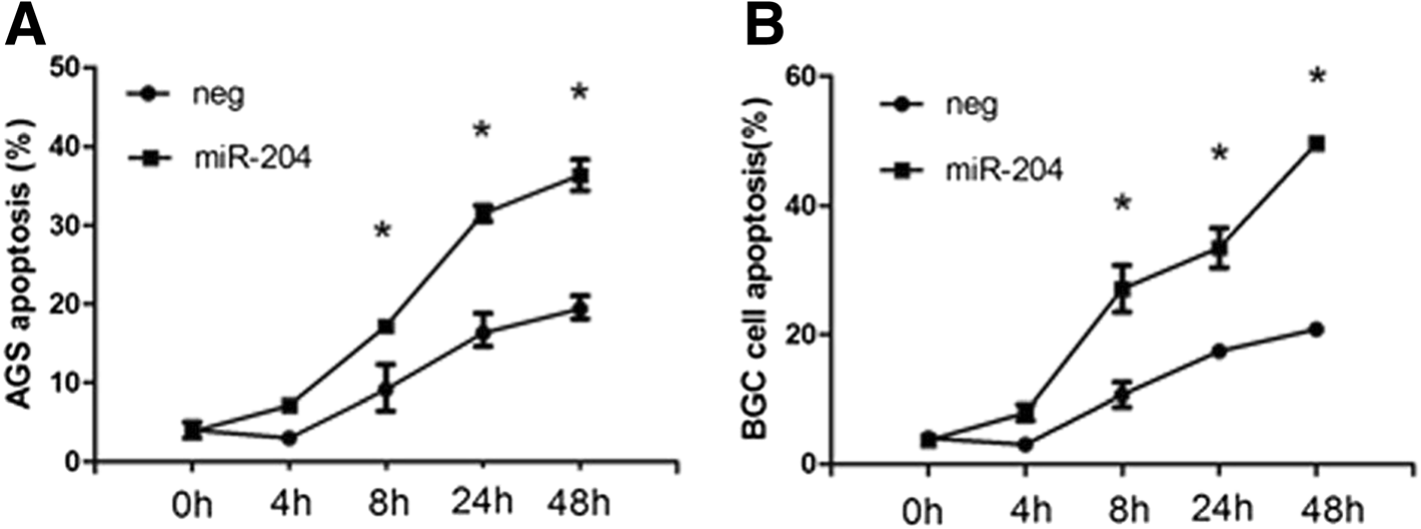
**MiR-204 increases sensitivity to anoikis.** AGS (**A**) and BGC (**B**) cells were transfected with miRNA constructs and plated on poly-HEMA coated plates. Cells were collected for apoptosis analysis by FACS analysis of Annexin V/PI stained cells. Columns mean of three biological replicates, bars, standard deviation of the mean. ANOVA * P < 0.05.

Given the known role of anoikis resistance association with EMT, we investigated the effect of miR-204 and SIRT1 on anoikis by performing FACS analysis of Annexin V/PI stained cells. In these assays, the cells were placed on poly-HEMA coated plates, which prevent them from adhering. The cells were forced to float in suspension until harvested for analysis. In the cell apoptosis analysis, miR-204 restoration and consequent SIRT1 knock-down resulted in increased cells positive for Annexin V/FITC staining, indicating an increase in apoptosis in these samples (Figure 6). Thus, restoration of miR-204 and knock-down of SIRT1 decreased anoikis resistance as indicated by a decrease in the viability of suspended cells and a concurrent increase in apoptosis.

### Down-regulation of miR-204 promotes cancer cell invasion by activating LKB1 through SIRT1

**Figure 7:**
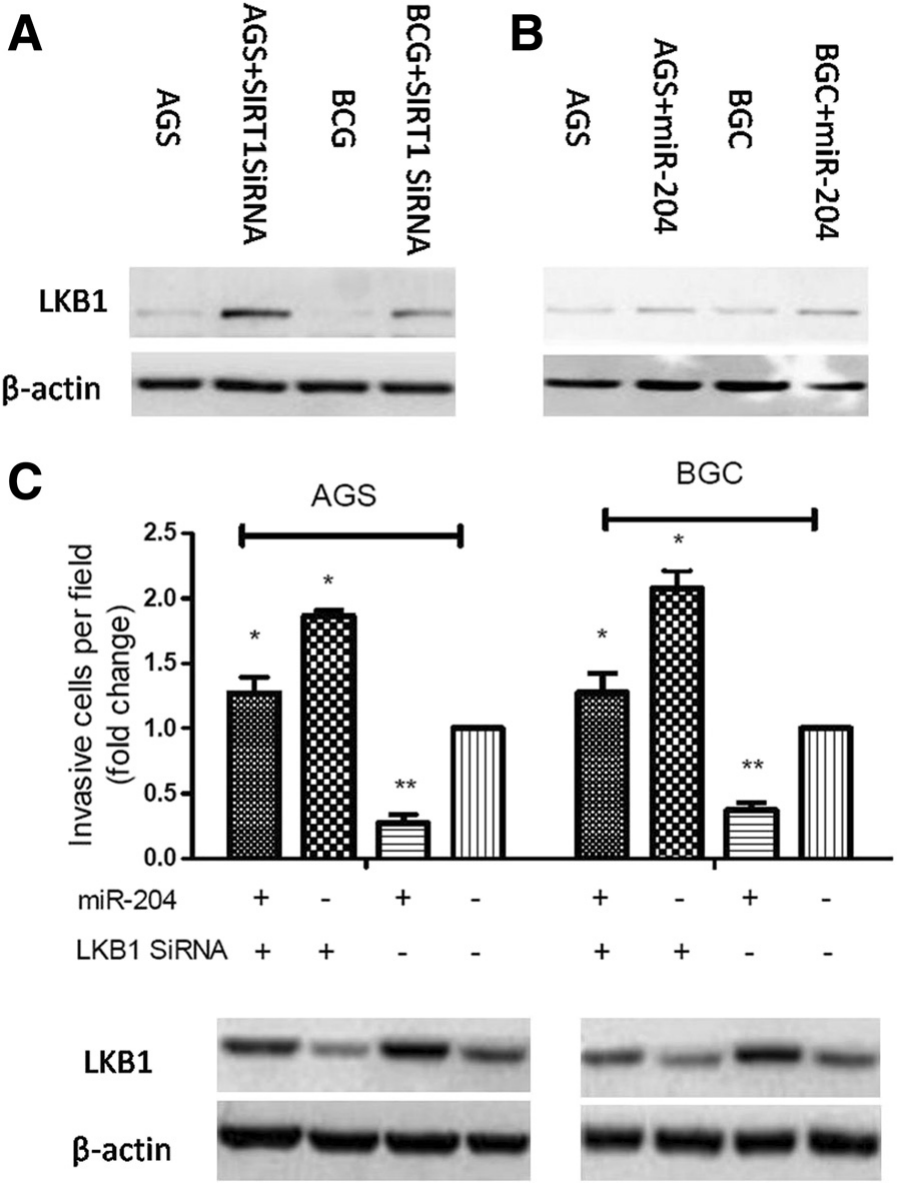
**MiR-204 activates LKB1 by repressing SIRT1expression.** (**A**) Western blot analysis showed the level of the endogenous LKB1 protein in AGS and BCG cells that were transfected with SIRT1 SiRNA. (**B**) Western blot analysis showed the level of endogenous LKB1 protein in AGS and BCG cells that were transfected with miR-204. (**C**) Inhibition of LKB1 could sabotage the miR-204 induced-cancer cell migration reduction. The profiles are representative of at least three independent experiments. N.C, negative control. *P < 0.05, **P<0.01.

LKB1 has a particularly tight link with EMT and anoikis. Therefore, we investigated the role of miR-204 in regulating LKB1. Western blot analysis of GC cells treated with miR-204 showed that miR-204 overexpression significantly increased LKB1 accumulation (Figure 7A). GC cells were transfected with SIRT1 SiRNA. Figure 7B shows that SIRT1 knockdown cells had a significant increase in LKB1 expression. AGS and BGC cells were co-transfected with either the LKB1 SiRNA or the negative control, along with either the miR-204 mimic or the microRNA control. Cells transfected with miR-204 mimics along with the negative control showed significantly decreased cell invasion ability compared with the two LKB1 SiRNA transfected groups(*P<0.05). LKB1 protein expression was negatively correlated with the cell invasion ability (Figure 7C). These data suggest that decreased expression of miR-204 might promotes cell metastasis by inactivating LKB1.

## Discussion

Class III histone deacetylase SIRT1 blocks senescence and apoptosis and promotes cell growth and angiogenesis, making it a critical regulator of tumor initiation, prognosis and drug resistance. Our previous studies have suggested that the up-regulation of SIRT1 is related to lymph node metastasis in gastric cancer [12]. The underlying mechanism by which this occurs is still unclear. There are 34 miRNAs are predicted to target the 3’UTR of SIRT1, which is 1.7 kb. We evaluated and analyzed the expression of these miRNAs that are conserved across various species in normal gastric mucosa tissue, gastric cancer specimens, and 2 gastric cancer cell lines. The results showed that reduced expression of miR-204 frequently occurred in gastric cancer tissues and was related with the up-regulation of SIRT1. We also verified this result in 24 gastric cancer tissues and found that the decreased expression of miR-204 was related with cancer metastasis.

miRNAs are involved in several important biological events such as tumorigenesis and cancer metastasis. miRNAs are known to act as regulators in gastric cancer cell growth and to regulate gastric cancer metastasis. Deregulation of some miRNAs, including miR-101, miR-107, miR-221, and miR-222, has been observed in gastric cancer [13-15]. miR-101 was down-regulated in gastric cancer tissues. The ectopic expression of miR-101 significantly inhibited cellular proliferation, migration, and invasion of gastric cancer cells by targeting EZH2, Cox-2, Mcl-1, and Fos. miR-107 is frequently up-regulated in gastric cancers, and its overexpression is significantly associated with gastric cancer metastasis. Here, we demonstrated miR-204, another miRNA specific to gastric cancer metastasis, and its specific target, SIRT1.

Epithelial–mesenchymal transition (EMT) consists of a rapid and often reversible change of cell phenotype [16]. Epithelial cells loosen cell–cell adhesion structures, including adherens junctions and desmosomes, to modulate their polarity and rearrange their cytoskeleton. Specifically, intermediate filaments typically switch from cytokeratins to Vimentin [17,18]. Cells become isolated, motile and resistant to apoptosis. Many genes and pathways have been implicated in inducing EMT in tumor cells. Typically, these pathways are also active in other processes, including cell proliferation, apoptosis and differentiation during early developmental stages, tissue morphogenesis and wound healing. Their specific role during human tumor progression is usually not well understood [19]. Our previous analysis of the clinical characteristics indicated that SIRT1 expression was significantly associated with tumor stage and the presence of metastasis, which further indicated that SIRT1 acts as a tumor promoter and facilitates the infiltration of gastric cancer. The oncogenic epithelial-to-mesenchymal transition (EMT) is thought to play an important role in tumor progression. Our current results suggest that miR-204 down-regulation and SIRT1 restoration can induce EMT in GC cells.

There is a tight correlation between anoikis resistance and oncogenic EMT [20-22]. A common hallmark of EMT is the breakdown of E-cadherin expression or function [23], which suffices to circumvent anoikis in some contexts. For example, the targeted knockout of the E-cadherin gene in a mouse mammary tumor model or the stable knockdown of E-cadherin in a mammary epithelial cell line confers anoikis resistance [24]. This finding implies that EMT-promoting transcription factors such as ZEB1/2, Snail1/2 and Twist can block anoikis both by directly regulating apoptosis control genes and by suppressing E-cadherin expression. Here, we discuss the mechanism by which E-cadherin suppression triggers signaling events that control other apoptosis regulatory genes [25]. This study also investigated whether the miR-204-SIRT1 pathway was involved in anoikis resistance and metastasis promotion in GC cells. We demonstrate that miR-204 down-regulation and SIRT1 overexpression both can induce anoikis resistance in GC cells.

LKB1 was identified originally as the tumor suppressor gene on human chromosome 19p13. LKB1 inactivation triggers EMT in lung cancer cells through the induction of ZEB1 [26]. Cheng et al. reported that LKB1 was an essential upstream regulator of p53-mediated anoikis [27]. Recent studies have revealed that many proteins, such as SIRT1, are involved in the regulation of LKB1 [28]. Over-expression of LKB1 promoted cellular senescence and retarded endothelial proliferation, which could be blocked by increasing SIRT1 levels. Knocking down of SIRT1 induced senescence and elevated the protein levels of LKB1. SIRT1 antagonized LKB1 activation through promoting deacetylation, ubiquitination and proteasome-mediated degradation of LKB1. Our data show that over-expression of miR-204 increased LKB1 expression in GCCs, while down-regulation of SIRT1 can also restore LKB1 expression in GCCs. LKB1 down-regulation could promote cancer cells invasion even when miR-204 was upregulated. As a result, miR-204 may modulate LKB1 by interacting with SIRT1. These data suggest that reduction of miR-204 promotes EMT by inactivating LKB1, and SIRT1 might be the direct target of miR-204 in the LKB1 pathway.

## Conclusion

In conclusion, our data demonstrate that the down-regulation of miR-204 promotes gastric cancer cell metastasis by activating the SIRT1-LKB1 pathway. Therefore, we show that miR-204 is an important regulator in gastric cancer metastasis and suggest the potential application of miR-204 in gastric cancer therapy.

## Competing interests

The authors declare that they have no competing interests.

## Authors’ contributions

LZ was responsible for planning the study. XW carried out the molecular genetic studies and involved in all steps of the data analysis and manuscript writing. PC provided laboratory support and helped to draft the manuscript. All authors read and approved the final manuscript.

## Pre-publication history

The pre-publication history for this paper can be accessed here:

http://www.biomedcentral.com/1471-2407/13/290/prepub
